# Unintended consequences: food prices increase in an Arctic indigenous community amidst road infrastructure development and loss of federal freight subsidy

**DOI:** 10.3389/fnut.2025.1521800

**Published:** 2025-05-16

**Authors:** Kenny Tiff-Annie, Li Nicholas, Mathieu Kim, Teddy Charmaine, Wesche Sonia, MacLean Jullian, Wolki Celina

**Affiliations:** ^1^Département de Médecine Sociale et Préventive, Université Laval, Québec, QC, Canada; ^2^CHU de Québec – Université Laval, Axe Santé des Populations et Pratiques Optimales en Santé, Québec, QC, Canada; ^3^Department of Economics, Toronto Metropolitan University, Toronto, ON, Canada; ^4^Department of Geography, Environment and Geomatics, University of Ottawa, Ottawa, ON, Canada; ^5^Community of Tuktoyaktuk, Tuktoyaktuk, NT, Canada; ^6^Inuvialuit Regional Corporation, Inuvik, NT, Canada; ^7^Inuvialuit Regional Corporation, Paulatuk, NT, Canada

**Keywords:** food security, food affordability, nutrition policy, subsidy programs, northern Canada, retail food environment, rural, remote

## Abstract

**Introduction:**

Road infrastructure development is often promoted as a strategy to reduce food insecurity in remote Arctic communities. However, the real-world impacts of such investments—particularly when tied to the withdrawal of food access supports such as the Nutrition North Canada (NNC) freight subsidy, which is provided to retailers to offset high transportation costs—remain poorly understood. This study examines the effects of the Inuvik–Tuktoyaktuk Highway opening and the concurrent loss of the NNC freight subsidy on food prices in Tuktoyaktuk, Northwest Territories.

**Methods:**

We used a quasi-experimental difference-in-differences design comparing Tuktoyaktuk (intervention community) to Aklavik (control community). Data sources included participatory food costing surveys, Northwest Territories Community Price Index data, and retailer-reported Nutrition North Canada price data. Analyses assessed trends in the Revised Northern Food Basket cost and individual product prices over time, using both aggregate and dynamic difference-in-differences models.

**Results:**

Prior to the opening of the Inuvik–Tuktoyaktuk Highway in November 2017, food prices in Tuktoyaktuk and Aklavik were closely aligned. Following the road’s construction and the withdrawal of full Nutrition North Canada subsidies for Tuktoyaktuk, prices in the community began to rise. Although prices initially remained stable, a significant divergence emerged over time: by March 2022, food prices in Tuktoyaktuk were nearly 20% higher than in Aklavik. Improved surface access did not reduce the price of non-subsidized goods, as anticipated. Freight savings, if realized, were minimal or not consistently passed on to consumers. The principal driver of food price increases appears to be the loss of ongoing freight supports, particularly as neighboring Aklavik benefitted from new NNC enhancements that Tuktoyaktuk no longer accessed.

**Conclusion:**

Infrastructure alone is insufficient to improve food affordability in remote Indigenous communities. Retailer pricing discretion and market dynamics may mediate the impacts of transportation investments. To avoid exacerbating food insecurity, infrastructure projects must be accompanied by dynamic monitoring, community-driven food security strategies, and policy frameworks based on real affordability metrics rather than proxy indicators like road access. Strengthening both market and country food systems is critical to supporting Indigenous food sovereignty and resilience.

## Introduction

Globally, road infrastructure development is widely promoted as a strategy to stimulate economic growth, improve market integration, enhance food access, and reduce living costs in rural and remote regions (1–3). Yet the impacts of these investments are highly context-dependent and often fall short of expectations. This is especially true in Indigenous communities, where economies and food systems are deeply interwoven with land-based livelihoods, cultural identity, and subsistence practices—and where greater access to market systems does not necessarily translate into improved food security or overall community well-being (4–8).

In the North American Arctic, road construction is accelerating after decades of infrastructure deficits, with dramatic changes in connectivity anticipated over the coming decades (9). These developments unfold alongside ongoing social, economic, and dietary transitions that Arctic Indigenous communities have experienced since government-mandated settlement in the mid-20th century. In Inuit Nunangat, these shifts have fundamentally reshaped the food system. Communities have increasingly navigated integration into wage economies and market-based food systems—often under inequitable and externally imposed terms—while continuing to uphold Inuit food systems rooted in harvesting, intergenerational knowledge, and cultural responsibility.

Today, store-bought foods represent a significant, if imperfect, component of everyday diets. While they offer convenience and year-round availability, they are often expensive, less nutritious, and poorly suited to northern contexts. At the same time, access to country foods is constrained by high harvesting costs, regulatory and policy barriers, and climate-related disruptions—further limiting the ability of households to meet food needs through traditional means. This results in a dual burden: dependence on nutritionally limited and high-cost market foods, alongside constrained access to culturally and nutritionally vital country foods.

This burden has placed enormous pressure on household food budgets across Inuit Nunangat. Market food prices are frequently two to three times higher than the national average (10, 11), while median household incomes remain approximately 30% below the Canadian average (12). Very low-income households are often unable to afford even a basic nutritious diet, covering as little as 6–13% of the cost of a healthy food basket (13). As a result, Inuit communities face some of the highest documented rates of food insecurity among Indigenous populations in high-income countries, with the majority of households affected (13, 14).

In response, policy efforts have largely focused on reducing the cost of store-bought foods, often through market-based frameworks that insufficiently reflect the complexity of Inuit food systems. The federal Nutrition North Canada (NNC) program—the cornerstone of Canada’s food access strategy in the North—provides freight subsidies to retailers for transporting perishable, nutritious foods into communities without year-round road access. NNC replaced the earlier Food Mail Program, which similarly limited eligibility to fly-in communities and aimed to reduce food costs by subsidizing air freight. While NNC has undergone several reforms—including the addition of a Harvesters Support Grant intended to bolster country food access—its central mechanism remains a freight subsidy aimed at lowering the price of market foods.

In parallel, investments in physical infrastructure—particularly road construction—have been increasingly framed as a long-term solution to food insecurity in the North (5, 15, 16). Improved road access is presumed to enable more reliable and cost-effective shipping, reducing dependence on expensive, weather-dependent air freight. This presumption is embedded in NNC program design itself: communities that gain permanent road access become ineligible for the full freight subsidy, on the assumption that surface transport will generate sufficient cost relief. Yet this assumption remains largely untested in Arctic contexts.

Expectations that road development will meaningfully reduce food prices rest on a chain of uncertain assumptions: that surface access will substantially lower freight costs; that retailers will pass those savings on to consumers; and that such reductions will be large enough to offset the withdrawal of NNC subsidies. In practice, food pricing in Arctic communities is shaped not only by transportation costs but by a broader constellation of structural and logistical factors—including limited retail competition, high operating and warehousing costs, low sales volumes, and the complexities of supply chain coordination in remote and climate-vulnerable environments. These constraints can blunt the impact of infrastructure investments, limiting the degree to which improved connectivity translates into lower shelf prices. As such, it remains unclear whether road development delivers net gains for food security—or whether, in some cases, it may displace critical policy supports like NNC without delivering equivalent benefits for households.

Beyond freight logistics, road infrastructure may also shape food access by influencing consumer behavior. According to household production theory, improved transportation can reduce the “full price” of food by lowering the time and effort required to travel for groceries—potentially increasing demand in destination markets and exerting upward pressure on prices (17). However, the relevance of this mechanism in Arctic and sub-Arctic contexts remains poorly understood. Our team is currently investigating these dynamics through qualitative research and a household cost-of-living survey, with the aim of better capturing evolving consumer access patterns in the region.

This study aims to empirically examine the impact of road development on food costs in the Inuvialuit Hamlet of Tuktoyaktuk, which gained year-round road access for the first time with the opening of the Inuvik-Tuktoyaktuk Highway in November 2017. The opening of the highway directly triggered the withdrawal of full NNC freight subsidies for Tuktoyaktuk, as eligibility for the program is contingent on the absence of year-round surface access. As a result, the effects of infrastructure development and freight subsidy loss in this case were inherently intertwined. The impetus for this study emerged from concerns raised by community members in Tuktoyaktuk during a 2018 food security engagement process, as residents questioned the trajectory of local food prices following the arrival of road access. These concerns contrasted with earlier official expectations. A 2010 territorial economic analysis projected that the highway would substantially reduce freight costs—from approximately $6.61/kg by air to $0.33/kg by road—and eliminate $456,000 in annual federal air freight subsidies under the Food Mail program, the precursor to NNC (18). While these anticipated savings were framed as benefits for both governments and consumers, their downstream impacts on food affordability remained unevaluated.

This study provides a rare opportunity to assess whether those expectations materialized in practice. Informed by both policy assumptions and community concerns at the time of the road’s opening, we framed our evaluation around three guiding hypotheses:

- H1: That improved surface access, and the initial loss of NNC subsidies, would have a net neutral or beneficial effect on food prices by reducing freight costs.
- H2: That surface access would at minimum reduce the cost of non-subsidized goods, such as shelf-stable and non-perishable items, both by enabling cheaper and more rapid transportation compared to air freight, and by reducing the frequency of costly emergency shipments when seasonal inventories proved insufficient.
- H3: That the loss of future NNC subsidy enhancements would not significantly affect long-term trends in food affordability.

Using a quasi-experimental difference-in-differences design, we critically examine whether infrastructure improvements sufficiently reduced food prices to compensate for the removal of federal assistance—or whether they inadvertently introduced new challenges to food affordability and the local food environment in Tuktoyaktuk.

## Methods

**Figure 1:**
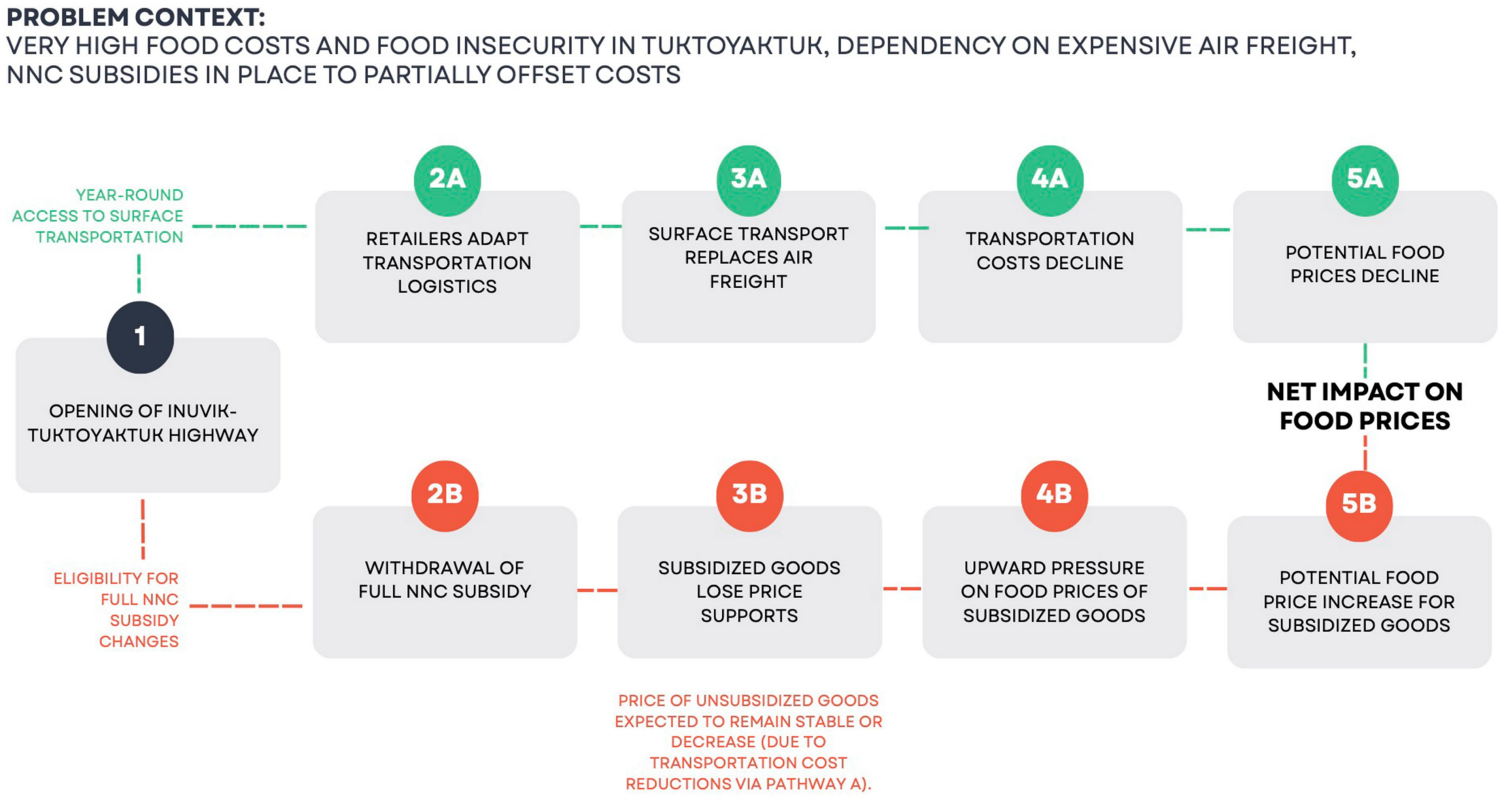
Conceptual pathways linking infrastructure and policy changes to food prices in Tuktoyaktuk.

We developed a conceptual pathways framework to illustrate the anticipated relationships between infrastructure development (the opening of the Inuvik–Tuktoyaktuk Highway), changes in NNC subsidy eligibility, and impacts on food affordability. Figure 1 summarizes the core assumptions guiding the study, mapping how shifts in transportation logistics, freight costs, and freight subsidy policies were expected to influence food prices and the local food environment.

### Study site

#### Community of Tuktoyaktuk

**Figure 2:**
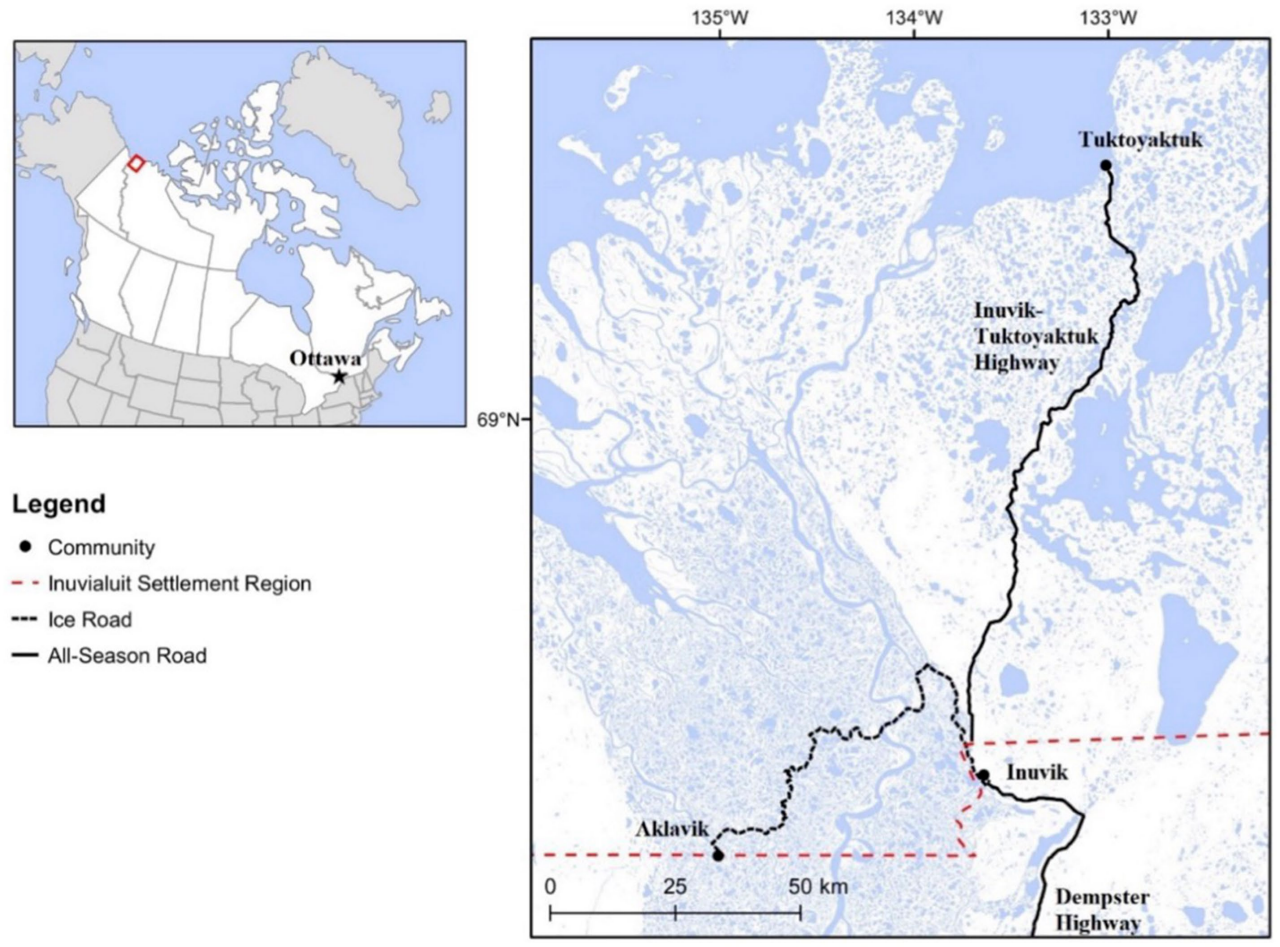
Map illustrating the Inuvik-Tuktoyaktuk Highway, connecting the Inuvialuit hamlet of Tuktoyaktuk to Inuvik and the rest of the continental road network via the Dempster Highway.

This study is based in the community of Tuktoyaktuk, located within the Inuvialuit Settlement Region (ISR) in the western Canadian Arctic. Tuktoyaktuk is part of the homeland of the Inuvialuit people, and its geographical context is shown in Figure 2. The ISR covers an area of 1,172,749 km^2^ and is home to 5,924 residents across six communities. Inuvik, the administrative center of the region, has year-round road access since the completion of the Dempster Highway in 1979, though access is periodically interrupted during seasonal thawing and freezing.

As of 2022, Tuktoyaktuk has a population of 1,058, with 987 residents identifying as Indigenous (19). The community’s economic structure is deeply intertwined with traditional activities with over half of the residents (55.4%) engaging in hunting and fishing and a similar percentage (55.3%) in gathering berries. Nearly a third (30.5%) contribute to the local economy through the production of arts and crafts. There is a strong dependency on subsistence activities, with a significant majority of households (60.2%) relying on country food for at least half of their dietary needs (19). Economic challenges are a significant concern in Tuktoyaktuk, with 21.9% of residents struggling with rent or mortgage payments due to rising costs (19). Food security is also a critical issue; in 2017, 43.8% of the population expressed concerns about their ability to afford food. While no updated local data on food security is available post-pandemic, the community continues to face challenges with high food prices, limited access to healthy and affordable food, and disruptions caused by climate change. Tuktoyaktuk is served by two grocery retailers—Stanton’s (Inuvialuit Development Corporation) and the Northern Store (Northwest Company). Before the road was built, Tuktoyaktuk’s market food supply was predominantly transported by air throughout the year. During the winter months, additional supplies were delivered via ice road, while non-perishable items were shipped by barge along the Mackenzie River during the summer months.

#### Opening of the Inuvik-Tuktoyaktuk highway

On November 15, 2017, the Inuvik-Tuktoyaktuk Highway (ITH) was officially opened, marking the first land-based connection to the Arctic Ocean in Canada. This 138 km highway, constructed at a cost of CAD$299 million and requiring an estimated CAD$1.9 million for annual maintenance, represents a significant engineering achievement in one of the world’s most challenging climates (18). The highway extends from Inuvik, where the Dempster Highway ends, providing direct access to the community of Tuktoyaktuk, located on the Arctic coast (18).

The ITH has been hailed as a transformative project, expected to drive job creation, enhance regional connectivity, and improve access to essential services, including food, housing, and fuel. These changes were anticipated to reduce living costs and significantly improve the quality of life for Tuktoyaktuk’s residents (4, 18).

However, the extent to which these benefits have materialized, particularly in relation to food prices, remains largely unexamined. While projections suggested that year-round road access would replace more expensive air and barge transport, few empirical assessments have evaluated the downstream impacts on the local cost of living. This study contributes to filling that gap by critically examining whether the ITH delivered the expected improvements in food affordability for Tuktoyaktuk households.

### Analytical approach

To evaluate the impact of the Inuvik-Tuktoyaktuk Highway on food prices in Tuktoyaktuk, we examine changes in the costs of a standard food basket—the Revised Northern Food Basket (RNFB)—as well as a broader set of individual food items. The additional set includes both subsidized and non-subsidized products, allowing us to assess whether the withdrawal of Nutrition North Canada subsidies and the introduction of year-round road access differentially impacted the affordability of various goods.

The RNFB was created by Indigenous and Northern Affairs Canada in collaboration with Health Canada. It consists of 67 food items that are designed to reflect the purchasing patterns of a family of four (1 man and 1 woman aged 25–49 years, 1 boy aged 13–15 years, and 1 girl aged 7–9 years, for 1 week). Most (about 85%) of the foods in the RNFB are subsidized by NNC. The RNFB is not intended as a nutrition guideline, does not meet all the recommended nutrient intakes of the Canadian Food Guide, and may not be representative of actual food consumption habits or expenditures in the populations concerned (10, 11, 20).

Aklavik was selected as a control community due to its demographic, economic, and logistical similarities to Tuktoyaktuk prior to the highway’s completion, including reliance on seasonal ice road access and comparable food pricing (Table 1). Both communities are served by the same major retailers—Stanton’s (operated by the Inuvialuit Regional Corporation) and the Northern Store (Northwest Company).

**Table 1:** Inuvialuit communities of Tuktoyaktuk, Aklavik, and Inuvik.

Variable	Tuktoyaktuk	Aklavik	Inuvik
Population (2016)	870	585	3,243
Median income (2015)	21,984	22,000	51,136
Mean income (2015)	37,569	36,255	66,050
Quarterly subs. KG shipped per capita (2017)	38.9	40.4	N/A
Level 1 freight subsidy per KG (2017)	2.60	1.60	Partial seasonal subsidy*
Level 2 freight subsidy per KG (2017)	0.70	0.05	N/A
RNFB freight subsidy (2017)	100	62	N/A
RNFB cost (2017)	427	433	N/A
Retailers	NWC, Stanton’s	NWC, Stanton’s	NWC, Stanton’s, several small convenience stores

Population and income from the 2016 Canadian Census. RNFB cost and quarterly subsidized shipments (KG per capita) from NNC website and freight subsidy from author’s calculations. * Community eligible for NNC subsidy during seasonal periods of isolation (break up/freeze up on Dempster highway).

Inuvik was also used as a secondary referent community in a subset of analyses. Although Inuvik differs from Tuktoyaktuk in population size and retail competition, it shares retail linkages through the same grocery networks. Moreover, Inuvik’s year-round road access and limited Nutrition North subsidy eligibility (restricted to freeze-up and thaw periods) offer a complementary perspective on how permanent transportation access may influence local food prices.

### Data sources

We draw on three complementary data sources to assess changes in food prices following the opening of the Inuvik-Tuktoyaktuk Highway: 1. participatory food costing surveys conducted in the Inuvialuit Settlement Region (ISR); 2. regional food price data published by the Northwest Territories (NWT) Bureau of Statistics; and 3. retailer-reported price data collected through the NNC program.

#### Participatory food costing

Participatory food costing employs community researchers to collect data on local food prices, offering a detailed, ground-level view of market conditions (21–23). Our team, in collaboration with the Inuvialuit Regional Corporation, has used this approach since 2014 through intermittent phases of data collection, depending on funding availability. Further details on the methodology and findings from earlier phases of this research are available in Kenny et al. (10) and Kenny et al. (24).

Community researchers collected food price information from a pre-defined list of over 100 items. The list of food and beverages included in the costing study was derived from multiple sources: the RNFB (20), dietary recalls from the 2007–2008 HIS (25), feedback from community research assistants, and input from the ISR Regional Dietitian. Costing sheets for 2017–2020 were double-entered in Excel by two independent researchers at the University of Ottawa. An error rate of 1.25% was found and corrected between the first and second entries. Missing data, such as where an item was unavailable or where the local researchers’ notes were illegible, were imputed by averaging the price of the item in the previous and following season. When the price was missing for more than four seasons before and after the missing data point, a regional average was used. Of the items in the food basket in Tuktoyaktuk, there were a total of 64 (16%) missing items out of 402 items in the first follow-up period, and 50 (15%) missing items out of 335 items in the second follow-up period. A mix of outlier detection techniques and researcher judgment was used to identify outliers. In cases where significant outlier values could not be explained after further examination, they were removed from the dataset and imputed as missing values. There was one significant outlier in Tuktoyaktuk during the study period (1/737, 0.1%) and 13 significant outliers in the regional dataset (13/5,243, 0.25%). Summary results from the food costing study are summarized in Table 2 for Tuktoyaktuk and the other communities of the ISR (anonymized for community confidentiality).

**Table 2:** Average cost of the Revised Northern Food Basket (CAD$/week) in the six communities in the Inuvialuit Settlement Region.

Season	Community^*^					
	Tuktoyaktuk	Com. B	Com. C	Com. D	Com. E	Com. F
Fall14	$359.64	$440.39	$432.15	$475.03		$473.98
Win14	$416.56		$493.91	$500.72	$448.92	$523.95
Sum15	$407.13	$403.91	$468.95	$528.40	$465.92	$461.07
Win15	$423.40		$516.94	$470.87		$441.52
Fall17	$457.28	$505.23				
Spr18	$445.96	$435.23				
Sum18	$454.36	$463.39				
Fall18	$449.96	$481.35				
Spr19	$487.83	$477.81				
Fall19	$477.25		$516.59	$530.62	$528.27	
Win19	$484.67	$432.83		$543.40	$527.61	$464.26
N^†^	16	12	6	6	4	5
Average	$449.13	$458.26	$490.63	$508.17	$492.68	$472.94

Data collected seasonally. Missing values represent seasons then data was not collected due to logistical or budgetary constraints. * Community names have been coded to ensure the privacy of retailer market prices. Tuktoyaktuk, the community of study, is not coded. ^†^ In communities where there are two stores, data was sometimes obtained in more than one store per season. For privacy reasons, only the average of both stores, when applicable, is shown in this table. The N displayed here counts all the data points available by community.

#### Northwest Territories Community Price Index

Our analysis incorporates data on food prices from the Northwest Territories Bureau of Statistics, available through its public website. The NWT Food Price Index is conducted periodically and covers approximately 300 products sold across all communities. Prices are reported relative to Yellowknife as the reference point. For example, the 2019 NWT Food Price Index indicates that the cost of living in Tuktoyaktuk was 158% of the cost in Yellowknife, while Inuvik was at 159% (26). In addition, a quarterly price survey is conducted for a subset of products and communities. Average annual prices were available for the years 2015, 2018, 2019, 2020, and 2022. Notably, Tuktoyaktuk and Inuvik are included in this dataset, but Aklavik is not. Price trends for Aklavik were assessed separately using retailer-reported NNC data. The list of products tracked varies across survey years, with approximately a dozen items reported consistently over time. The NWT Bureau of Statistics advises caution when interpreting longitudinal trends due to potential inconsistencies in product selection and data collection timing. Although efforts are made to price consistent brands and package sizes year over year, substitutions occasionally occur. Furthermore, annual average prices are less suited for detecting mid-year policy changes—such as the opening of the Inuvik-Tuktoyaktuk Highway or the withdrawal of NNC subsidies—and the data are affected by missing quarters (e.g., Inuvik is missing 2018Q1, and Tuktoyaktuk is missing 2020Q1). These limitations complicate direct longitudinal comparisons.

#### Nutrition North Canada (NNC) retailer data

Our analysis also incorporates data from the NNC program, which has required northern retailers to report prices for specific food items since 2011. This government-mandated program monitors food affordability in communities eligible for federal freight subsidies using store-level pricing data. The NNC publishes quarterly aggregated data on the RNFB providing a standardized measure of food costs over time and across communities. We downloaded and used the final per-community RNFB costs published on the NNC website for our study period.

In addition to the aggregated RNFB data, we used confidential product-level price data that retailers submit to NNC as a condition of program participation. These data are reported at the store-by-month level and include a large set of individual barcodes. An external firm processes these data to match products to the RNFB list and assign quantities and weights. NNC officials validate prices for key reporting months (March, July, September, December), then aggregate, average, and impute values as necessary to produce the public RNFB estimates. In particular, we have access to the “cleaned” version of these matched data, which have been checked for outliers and were used directly by NNC to calculate public RNFB figures. These cleaned data are available quarterly through March 2021. We also have access to the unprocessed, original data files received by NNC, which are available monthly through March 2022 and include almost double the number of barcodes. Unprocessed data have not been fully verified; however, they offer an additional year of observation and finer monthly data resolution. In our main analyses, we focus on the cleaned and matched dataset but extend the analysis periods where appropriate.

We also provide supplemental analyses using the broader, unprocessed dataset. This allows us to examine a larger number of products, including items beyond the RNFB list (e.g., soda, bottled water). However, these broader data do not include verified product weights or NNC subsidy categorizations and may include more recording errors. For the RNFB-matched data, we assign freight subsidy eligibility and subsidy levels based on NNC program documentation over time. We also collected data on subsidy rate changes to account for the removal of subsidies in Tuktoyaktuk (March 2018) and the subsequent subsidy increases in Aklavik (January 2019 and May 2020). Subsidies are expressed in dollars per kilogram, which supports direct comparison with changes in $/kg prices but not percentage price changes.

### Difference-in-differences (DiD) analysis

To assess the impact of policy changes and infrastructure development on local food prices, we employ a difference-in-differences (DiD) approach using individual price data from the NNC program for both Tuktoyaktuk and Aklavik. This method allows us to control for both time-invariant and time-variant factors that might influence pricing, thereby isolating the effects of the interventions. Specifically, we analyze the impact of freight subsidy changes and the introduction of year-round surface access on food prices in Tuktoyaktuk relative to Aklavik.

Our regression model is specified as follows:

$$\text{Pric}e_{\text{irct}}=\alpha_{\mathrm{irc}}+\gamma_{\mathrm{irt}}+{\text{βPost}}_{t}*{\text{Tuktoyaktuk}}_{c}+\varepsilon_{\text{irct}}$$

Where:

- *i* indexes products,
- *r* indexes retailers,
- *c* indexes communities,
- *t* indexes time periods (either monthly or quarterly).
- In this model:
-
$\alpha_{\mathrm{irc}}$ represents fixed effects for each product-retailer-community combination, capturing any time-invariant characteristics that affect the price of a specific product in a specific retailer and community.
-
$\gamma_{\mathrm{irt}}$ represents fixed effects for each product-retailer-time combination, accounting for time-varying factors that affect a retailer’s pricing for a product that are common to both communities. This includes variables like wholesale costs and portions of freight and warehousing costs, given that products shipped to either community by the same retailer likely pass through common logistic hubs such as Inuvik.

The interaction term ${\text{βPost}}_{t}*{\text{Tuktoyaktuk}}_{c}$ captures the ‘treatment effect’ of interest—the change in prices for Tuktoyaktuk relative to Aklavik during the post-treatment period, defined as beginning in April 2018. This period coincides with the withdrawal of NNC subsidies and the loss of access to surface transport via ice roads (prior to 2018).

When limiting the analysis to periods up to January 2019, the DiD estimate isolates short-term effects associated with the simultaneous improvement in transportation and freight subsidy removal. When considering longer-term outcomes, the DiD estimate also reflects the effects of freight subsidy increases in Aklavik and other NNC-eligible communities in January 2019 and May 2020. To help distinguish these effects, we also estimate specifications with two additional post-treatment terms to capture the impacts of the NNC subsidy increases in Aklavik.

To further disentangle the impacts of freight subsidy changes and improved surface access, we provide separate estimates for groups of products by subsidy level. When subsidies were removed for Tuktoyaktuk in April 2018, two freight subsidy levels were in effect; a third, higher subsidy level was introduced in January 2019 for milk and frozen fruits and vegetables. Changes in price per kilogram for each group are compared to changes in subsidy amounts to provide a rough estimate of subsidy pass-through. Additionally, we analyze log-transformed prices to facilitate interpretation in percentage terms and to allow consistent comparison across different product categories.

We also estimate a “dynamic” DiD model by regressing product prices on a full set of time dummies interacted with the Tuktoyaktuk treatment. This allows us to assess the evolution of price changes over time and provides a robust test for any pre-existing differential trends between Tuktoyaktuk and Aklavik before the intervention. The dynamic model is specified as:

$$\text{Pric}e_{\text{irct}}=\alpha_{\mathrm{irc}}+\gamma_{\mathrm{irt}}+\sum_{k=1}^{T}\beta_{k}{\text{Period}}_{k}*{\text{Tuktoyaktuk}}_{c}+\varepsilon_{\text{irct}}$$

As in the baseline specification, we include product-retailer-community and product-retailer-time fixed effects.

Finally, to illuminate broader price trends, we estimate separate regressions of prices on time dummies for each community:

$$\text{Price}=\alpha_{\mathrm{irc}}+\sum_{k=1}^{T}\beta_{k}{\text{Period}}_{k}+\varepsilon_{\text{irct}}$$

where March 2018 serves as the omitted reference category.

## Results

This section presents our analysis of the impact of the Inuvik-Tuktoyaktuk Highway and the concomitant withdrawal of full NNC subsidies on food prices in Tuktoyaktuk. Drawing on multiple data sources, we examine trends in aggregate food price indices and individual food prices before and after these interventions. The results are interpreted in light of the three guiding hypotheses: (H1) that improved surface access, combined with the initial loss of NNC subsidies, would have a neutral or beneficial effect on food prices; (H2) that surface access would reduce the cost of non-subsidized goods; and (H3) that the loss of future NNC subsidy enhancements would not significantly affect long-term food affordability.

### Trends in aggregate food price indices across communities

**Figure 3:**
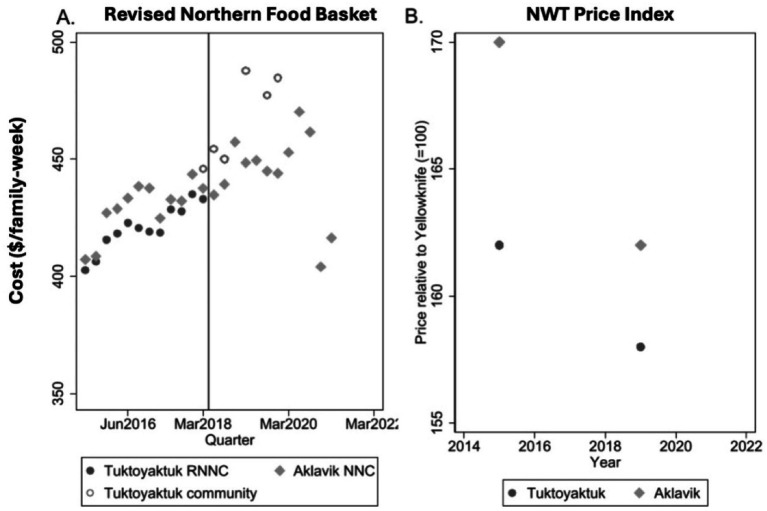
Trends in the cost of the Revised Northern Food Basket (RNFB) and food price index data for Tuktoyaktuk and Aklavik. **Panel A**: Trends in the cost of the Revised Northern Food Basket (RNFB) in Tuktoyaktuk and Aklavik from a participatory food costing study and Nutrition North Canada (NNC) reported data. **Panel B**: Northwest Territories (NWT) Food Price Index data for Tuktoyaktuk and Aklavik relative to Yellowknife.

Figure 3 displays trends in aggregate food price indices for Tuktoyaktuk and Aklavik, using three different sources. Panel A illustrates cost fluctuations in the Revised Northern Food Basket (RNFB) as published on the NNC website, supplemented by participatory food costing data collected in Tuktoyaktuk during 2018 and 2019. Panel B presents the Northwest Territories Food Price Index based on the Community Price Survey. Analysis of these data sources shows similar pricing patterns between Tuktoyaktuk and Aklavik during the pre-highway period. Following the withdrawal of subsidies and the opening of the highway in 2018, however, prices in Tuktoyaktuk began to diverge upward relative to Aklavik. Although the data points are limited, they suggest a noticeable relative increase in Tuktoyaktuk food prices post-2018, supporting further examination through a DiD framework.

Using the NWT Food Price Index and comparing the years 2015 to 2019, the DiD estimate implies a 4.7% relative increase in prices for Tuktoyaktuk. However, because these indices are benchmarked to Yellowknife, we cannot determine absolute price changes from these data alone. To provide a more detailed view, we merged RNFB cost data reported to NNC prior to 2018 with participatory food costing data collected afterwards in Tuktoyaktuk and compared this combined series to Aklavik’s RNFB costs as reported by NNC. This DiD analysis suggests a minimal short-term effect (an increase of a few dollars or a few percentage points within two quarters after March 2018), but a substantial longer-term effect: RNFB costs in Tuktoyaktuk rose by approximately $40–45, or around 10%, between March 2018 and Fall 2019. This long-term increase was driven primarily by rising prices in Tuktoyaktuk, even as Aklavik benefitted from an increase in NNC subsidy rates in January 2019.

The implied pre-2018 subsidy for shipping one RNFB to Tuktoyaktuk was approximately $100 overall and $92 for perishables. Following the loss of this freight subsidy, the observed $40–45 increase in RNFB price suggests a pass-through rate of about 40–50%, if considered in isolation. However, because transportation costs were also expected to decline following the opening of the highway—with projected freight savings of approximately $228 per RNFB [based on a drop from $6.61/kg by air to $0.33/kg by road (18)]—the observed price increase likely underestimates the full effect of freight subsidy loss. Taken together, these results suggest that while short-term projections of stable food prices were roughly accurate immediately after the road opening, longer-term outcomes reveal a dominant influence of freight subsidy withdrawal, rather than surface access improvements, in driving price increases in Tuktoyaktuk. Although initial price stability could suggest that freight cost reductions offset freight subsidy losses to some extent, emerging price gaps and retailer pricing behavior indicate that any savings may not have been consistently or sustainably passed through to consumers. These calculations also highlight the sensitivity of food prices to assumptions about freight costs: despite substantial projected freight savings, the loss of the air freight subsidy appears to have left the community more exposed to variability in surface transportation costs. Overall, these findings reject H1.

### Trends in individual food prices across communities

To further explore the pathways of price change, particularly whether surface access reduced the price of non-subsidized goods as anticipated under H2, we analyzed trends in individual food item prices across Tuktoyaktuk and Aklavik. We used two complementary sources: the NWT Community Price Survey and detailed confidential price data provided by retailers to NNC.

**Figure 4:**
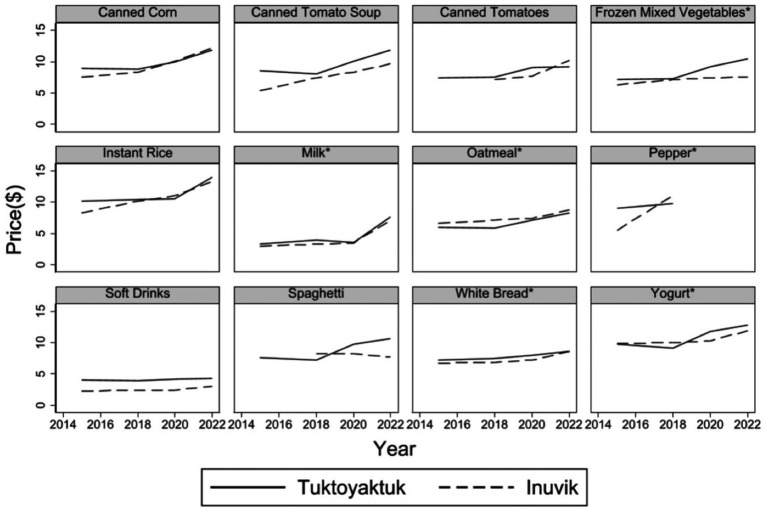
Comparison of product-level prices for Tuktoyaktuk and Inuvik based on data from the Northwest Territories Community Price Index. A star (*) indicates products that received a Nutrition North Canada (NNC) freight subsidy in Tuktoyaktuk prior to April 2018, amounting to $2.60 per kilogram.

Figure 4 shows price trajectories for a selection of food items reported through the Community Survey. While some items, such as canned corn and instant rice, exhibited price convergence between Tuktoyaktuk and Aklavik, others—such as frozen mixed vegetables, spaghetti, and yogurt—became relatively more expensive in Tuktoyaktuk after 2018. Although these trends suggest an emerging divergence post-subsidy withdrawal, the small number of common products and the limited precision in product matching limit the strength of this evidence.

**Figure 5:**
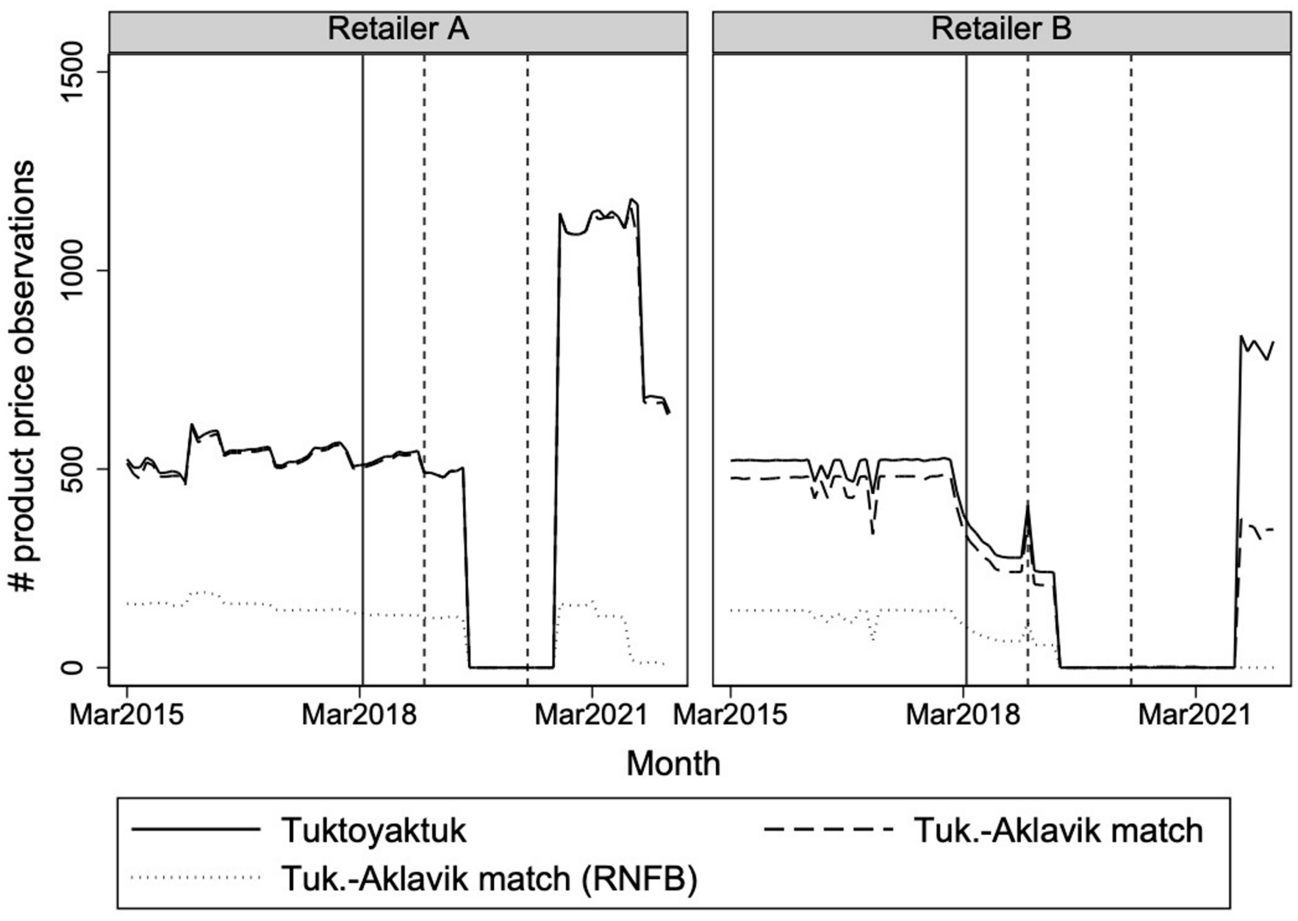
Trends in the number of store-product prices recorded for the two retail stores in Tuktoyaktuk, along with the subset of these products that match with identical store-products in Aklavik and the subset of these that directly enter the RNFB. The solid vertical line denotes the loss of NNC freight subsidy eligibility in Tuktoyaktuk and the two subsequent dashed vertical lines denote the timing of NNC subsidy increases for Aklavik in January 2019 and May 2020.

The NNC retailer-provided price data offer a richer and more precise dataset. Figure 5 displays the number of barcode-level products with available prices for Tuktoyaktuk, for Aklavik, and for those products used to calculate RNFB costs. Before April 2018, over 1,000 products were tracked in Tuktoyaktuk, with many matches in Aklavik. Following the loss of NNC eligibility, reporting dropped sharply, particularly between mid-2019 and 2021, though some reporting resumed during periods when Tuktoyaktuk received temporary NNC support during highway freeze-up.

**Figure 6:**
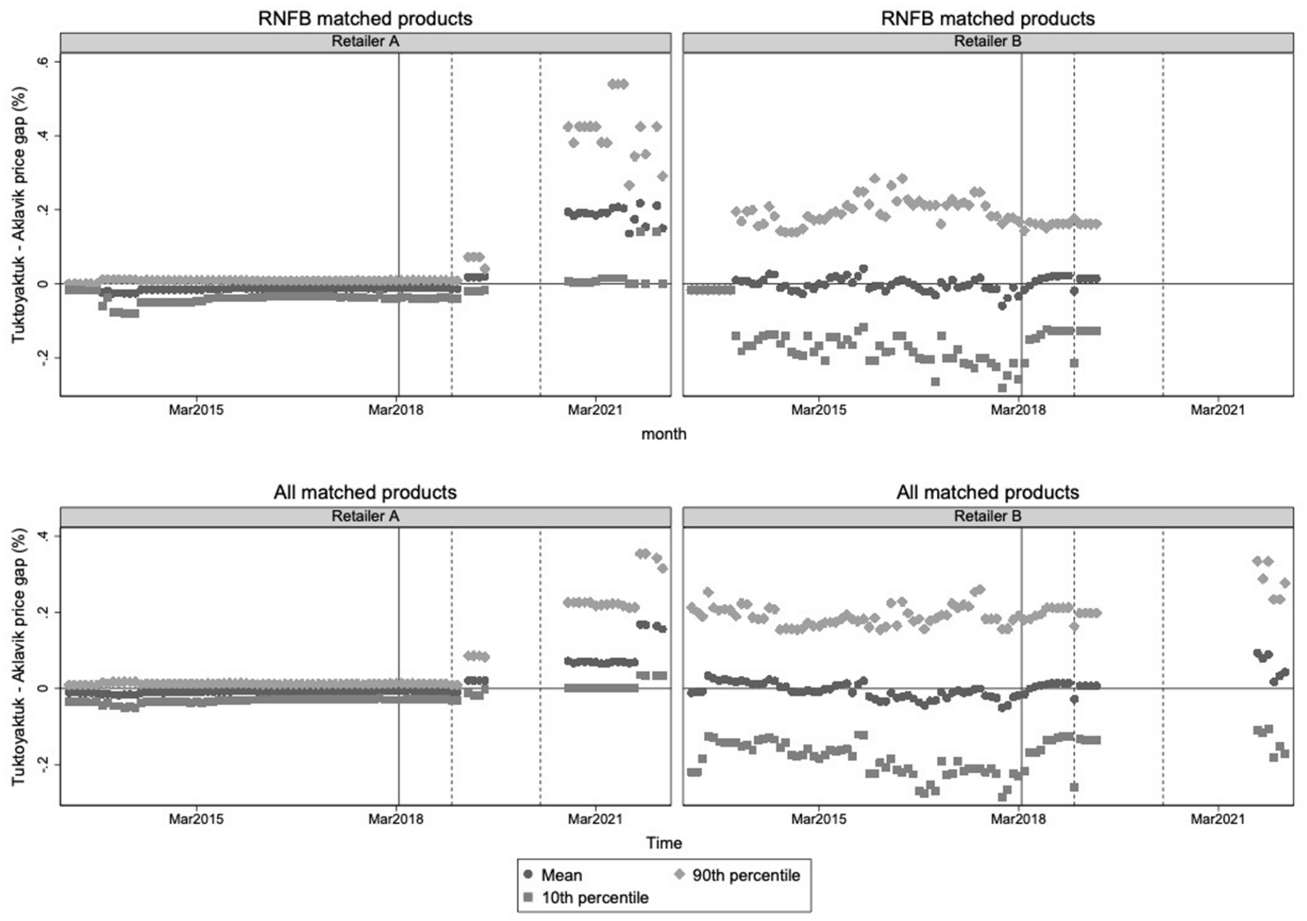
Price gaps for Tuktoyaktuk-Aklavik matched product-retailer Pairs, by retailer. Top panels present mean, 10th and 90th percentile price gaps, expressed in percent terms, for the RNFB matched products. Bottom panels present means, 10th and 90th percentile price gaps for all matched products. The solid vertical line denotes the loss of NNC freight subsidy eligibility in Tuktoyaktuk and the two subsequent dashed vertical lines denote the timing of NNC subsidy increases for Aklavik in January 2019 and May 2020.

Building on these data, Figure 6 presents the average log price ratio between Tuktoyaktuk and Aklavik across time. Before April 2018, average price differences between the two communities were negligible, suggesting that subsidies effectively equalized prices. Following the withdrawal of subsidies, price gaps widened, although increases remained modest until mid-2019. By 2020, average prices in Tuktoyaktuk were consistently 10–20% higher than in Aklavik, depending on the retailer. Importantly, no systematic reduction in the price of non-subsidized goods was observed, either in the short or long term. These findings are notable because there were plausible reasons to expect surface access would lower the cost of non-subsidized goods. First, year-round highway access likely allows faster and more efficient trucking compared to ice roads. Second, it reduces reliance on expensive emergency air freight shipments when seasonal stock runs low. The absence of price reductions even for unsubsidized goods suggests that cost savings were minimal or may not have been passed on to consumers. This also implies that broader classes of goods not directly observed in our dataset—including non-food items and household supplies—likely did not experience substantial price reductions either.

Figure 6 further illustrates that before April 2018, the distribution of price gaps between Tuktoyaktuk and Aklavik was centered tightly around zero, particularly for Retailer A, where the 90th and 10th percentiles of the price gap distribution were closely aligned. This suggests minimal variance in pricing across products and effective price equalization across communities prior to the highway opening and freight subsidy withdrawal. In contrast, Retailer B already exhibited greater dispersion before 2018, with many products displaying either positive or negative price gaps of around 20%. Following April 2018, the distribution of price gaps widened markedly for both retailers. After 2020, price gaps as large as 40% were observed for over one-tenth of the products with reported prices in each retailer, highlighting not only an overall rise in prices in Tuktoyaktuk relative to Aklavik but also increased variability in pricing strategies across individual food items.

The widening price gaps between Tuktoyaktuk and Aklavik, particularly after 2020, suggest that the combined effects of road development and the withdrawal of subsidies contributed to significant changes in the local food environment. To formally quantify these impacts and distinguish them from broader regional trends, we employ a DiD approach comparing price trajectories between the two communities.

### Difference-in-differences (DiD) analysis

To assess the impact of the Inuvik-Tuktoyaktuk Highway and the withdrawal of NNC subsidies on food prices in Tuktoyaktuk, we apply a DiD approach. This method compares changes in food prices in Tuktoyaktuk to those in Aklavik, which serves as a control community. In the main analysis, we present results using all available periods, although supplementary analyses (available upon request) show similar results when restricting to periods where public RNFB reporting is available.

Table 3 presents regression results capturing the DiD estimate (Tuktoyaktuk*Post) under various definitions of the post-treatment period. Columns 1 and 2 report results for prices in levels ($/kg) and in logarithmic terms, respectively, using a short-term sample ending in December 2018, before any changes in NNC subsidies for Aklavik. Columns 3 and 4 expand the sample to pool all available post periods. Columns 5 and 6 disaggregate the post period into three distinct phases: post-April 2018, post-January 2019, and post-May 2020, corresponding to the periods when freight subsidy policies changed for one of the communities. Changes in log prices can be interpreted approximately as percentage changes.

**Table 3:** OLS regression estimates of effect of Tuktoyaktuk highway completion and freight subsidy loss on Tuktoyaktuk prices (Aklavik control group).

Sample period	(1)	(2)	(3)	(4)	(5)	(6)
	Short-run (ends Dec.2018)		Long-run		Long-run	
Dependent variable	Price ($/KG)	Log(Price)	Price ($/KG)	Log(Price)	Price ($/KG)	Log(Price)
Post Apr. 2018 * Tuktoyaktuk	−0.010	−0.000	0.185***	0.035***	−0.008	−0.000
	(0.032)	(0.004)	(0.032)	(0.005)	(0.031)	(0.004)
Post Jan.2019 * Tuktoyaktuk					0.055**	−0.006
					(0.026)	(0.006)
Post May 2020 * Tuktoyaktuk					1.000***	0.224***
					(0.051)	(0.018)
Observations	36,332	36,332	41,493	41,493	41,493	41,493
Adj R-squared	0.992	0.986	0.992	0.985	0.992	0.987
Joint effect of all post*Tuktoyaktuk terms					1.046	0.218
*p*-value for test that joint effect = 0					0.000	0.000

All regressions include product-retailer-community and product-retailer-month fixed effects. Standard errors clustered by product-community in parentheses. *** *p* < 0.01, ** *p* < 0.05, * *p* < 0.10.

The results show that there was almost no change in the short term, with small and precisely estimated near-zero effects. Over the longer term, pooling all post periods, there is only a modest increase in Tuktoyaktuk prices equivalent to 18.5 cents per kilogram, or approximately 3.5%. However, this pooled estimate masks substantial differences between subperiods: relative prices remained stable until mid-2019 but began increasing significantly after 2020. In particular, columns 5 and 6 reveal large price increases in Tuktoyaktuk following May 2020, amounting to approximately $1 per kilogram or 22.4%. These findings indicate that the combined effects of improved surface transit and the loss of NNC eligibility resulted in significantly higher food prices for Tuktoyaktuk households within 3 years, with much of the long-term increase likely driven by the withdrawal of access to more generous subsidies introduced after 2020.

To further disentangle the contributions of freight subsidy changes and freight cost changes, Table 4 reports separate estimates for RNFB products grouped by subsidy eligibility: no subsidy, low subsidy, medium subsidy, and high freight subsidy levels (using the January 2019 NNC subsidy classifications). This analysis focuses on the long-term sample and separates the “post*Tuktoyaktuk” interaction into three post-period indicators.

**Table 4:** Heterogeneity: OLS regression estimates of effect of Tuktoyaktuk highway completion and freight subsidy loss on Tuktoyaktuk prices for different subsidy levels (Aklavik control group).

Freight subsidy level	(1)	(2)	(3)	(4)	(5)	(6)	(7)	(8)
	None		Low		Medium		High	
Dependent variable	Price ($/KG)	Log(Price)	Price ($/KG)	Log(Price)	Price ($/KG)	Log(Price)	Price ($/KG)	Log(Price)
Post Apr. 2018 * Tuktoyaktuk	−0.106	−0.010	0.149	0.019	0.034	0.003	−0.024	0.001
	(0.064)	(0.007)	(0.162)	(0.027)	(0.044)	(0.006)	(0.021)	(0.003)
Post Jan. 2019 * Tuktoyaktuk	0.035	0.005	0.392**	0.057**	0.086**	0.008	−0.051	−0.060***
	(0.043)	(0.004)	(0.156)	(0.027)	(0.035)	(0.007)	(0.031)	(0.017)
Post May 2020 * Tuktoyaktuk	0.048	0.004	0.544***	0.080***	1.044***	0.233***	1.389***	0.363***
	(0.054)	(0.005)	(0.093)	(0.019)	(0.049)	(0.021)	(0.040)	(0.038)
Change in Tuktoyaktuk vs. Aklavik freight subsidy ($/KG)								
Apr. 2018	0		−0.7		−2.6		−2.6	
Jan. 2019	0		−0.2		−0.95		−0.65	
May. 2020	0		−1		0		−0.65	
Observations	9,480	9,480	2,314	2,314	22,622	22,622	4,750	4,750
Adj R-squared	0.968	0.956	0.982	0.975	0.995	0.989	0.987	0.994
Joint effect of all post*Tuk. terms	−0.0230	−0.00213	1.086	0.156	1.165	0.244	1.313	0.304
*P*-value for test that joint effect = 0	0.143	0.185	0	4.33e-07	0	0	0	0

All regressions include product-retailer-community and product-retailer-month fixed effects. Standard errors clustered by product-community in parentheses. *** *p* < 0.01, ** *p* < 0.05, * *p* < 0.10. Freight subsidy levels are based on Jan. 2019 classification.

This heterogeneity analysis yields several important findings. First, there were no significant changes in the Tuktoyaktuk-Aklavik relative prices of RNFB products that were not eligible for NNC subsidies. Although freight subsidy changes were not expected to affect these products, it is notable that improvements in surface transport did not lead to lower prices even for non-perishable goods. Second, there was no detectable short-term effect of the highway opening and the concomitant withdrawal of subsidies on subsidized goods, suggesting that retailers maintained stable pricing regardless of the size of the subsidy lost. Products that lost medium or high subsidies (approximately $2.60/kg) exhibited similar near-zero price changes to products that lost only low subsidies (approximately $0.70/kg), implying no immediate re-pricing in response to freight subsidy withdrawal. Third, the long-term price increases in Tuktoyaktuk were closely correlated with the magnitude of relative subsidy changes, particularly the NNC subsidy increases received by Aklavik. Goods eligible for the highest freight subsidy level became $1.31/kg more expensive in Tuktoyaktuk relative to Aklavik, goods with medium subsidy eligibility became $1.17/kg more expensive, and goods with low subsidy eligibility became $1.09/kg more expensive. These results are consistent with full pass-through of subsidy increases to Aklavik consumers in the long term, and either zero or partial pass-through of NNC subsidy decreases in Tuktoyaktuk. Overall, the findings indicate that relative price changes between the two communities were primarily driven by differential subsidy changes across products.

**Figure 7:**
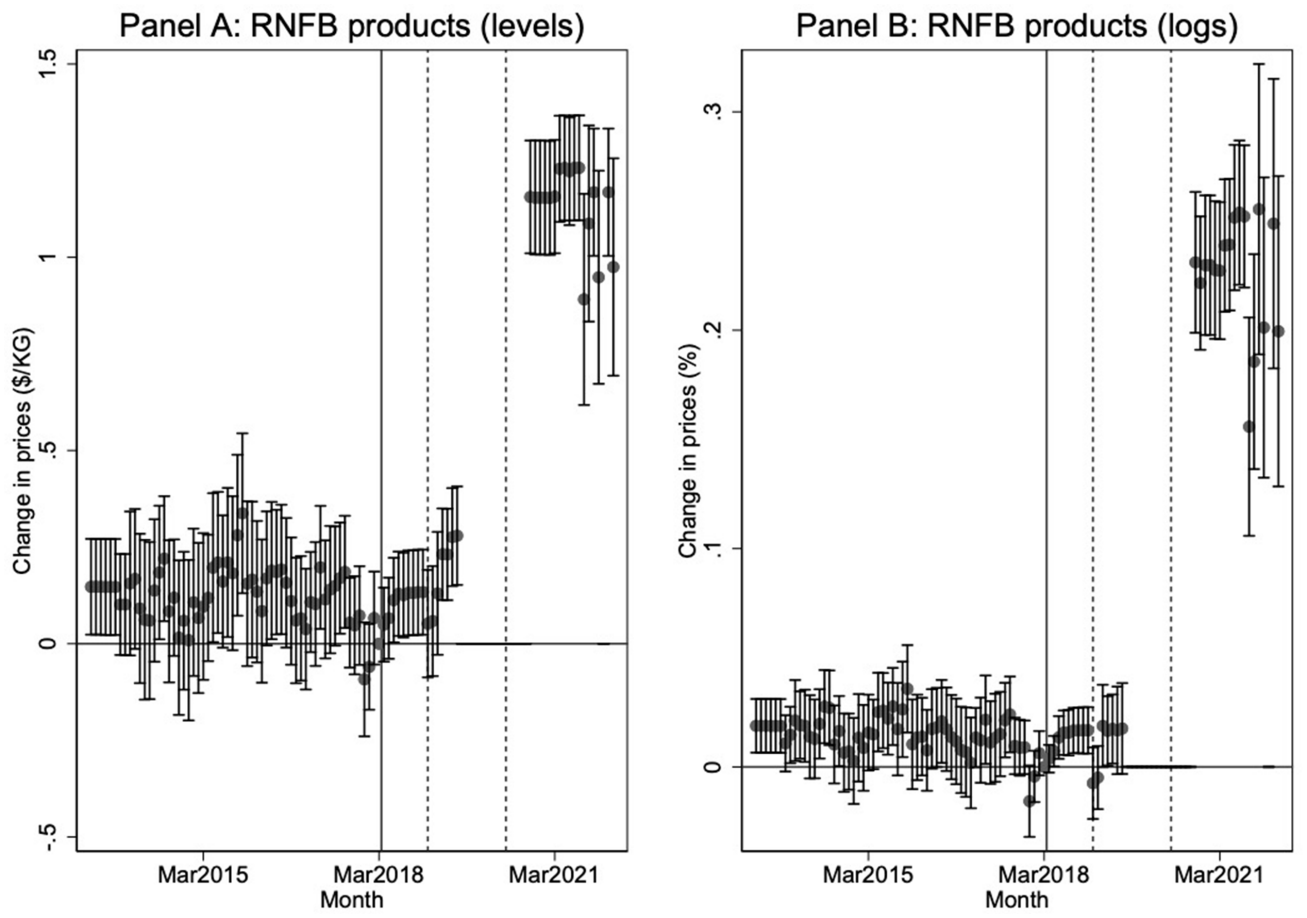
Dynamic difference-in-difference estimates of the effect of the Inuvik-Tuktoyaktuk Highway and loss of NNC eligibility on Product Prices. **Panel A**: Change in prices (levels, $/KG) for Revised Northern Food Basket (RNFB) products and all months of available data (relative to March 2018). **Panel B**: Change in prices (logs) for RNFB products and all months of available data (relative to March 2018). The solid vertical line denotes the loss of NNC freight subsidy eligibility in Tuktoyaktuk and the two subsequent dashed vertical lines denote the timing of NNC subsidy increases for Aklavik in January 2019 and May 2020.

Figure 7 provides an alternative presentation of the DiD estimates by plotting individual coefficients from an extended model that interacts community status (Tuktoyaktuk) with period dummy variables. March 2018 is used as the reference category, and the coefficients represent either dollar-per-kilogram or percentage changes in relative prices between Tuktoyaktuk and Aklavik for identical product-retailer pairs. The results in Panel A (price per kilogram) and Panel B (log prices) confirm the absence of any significant pre-treatment trends, strengthening the validity of the DiD identification strategy. Post-treatment effects, particularly after the withdrawal of subsidies in 2018 and the resumption of data reporting in fall 2020, reveal a persistent and substantial increase in Tuktoyaktuk prices relative to Aklavik, approaching 20% by late 2021. These results reinforce the interpretation that consumers in Tuktoyaktuk faced significantly higher costs for basic food items following the highway opening and withdrawal of NNC subsidies.

**Figure 8:**
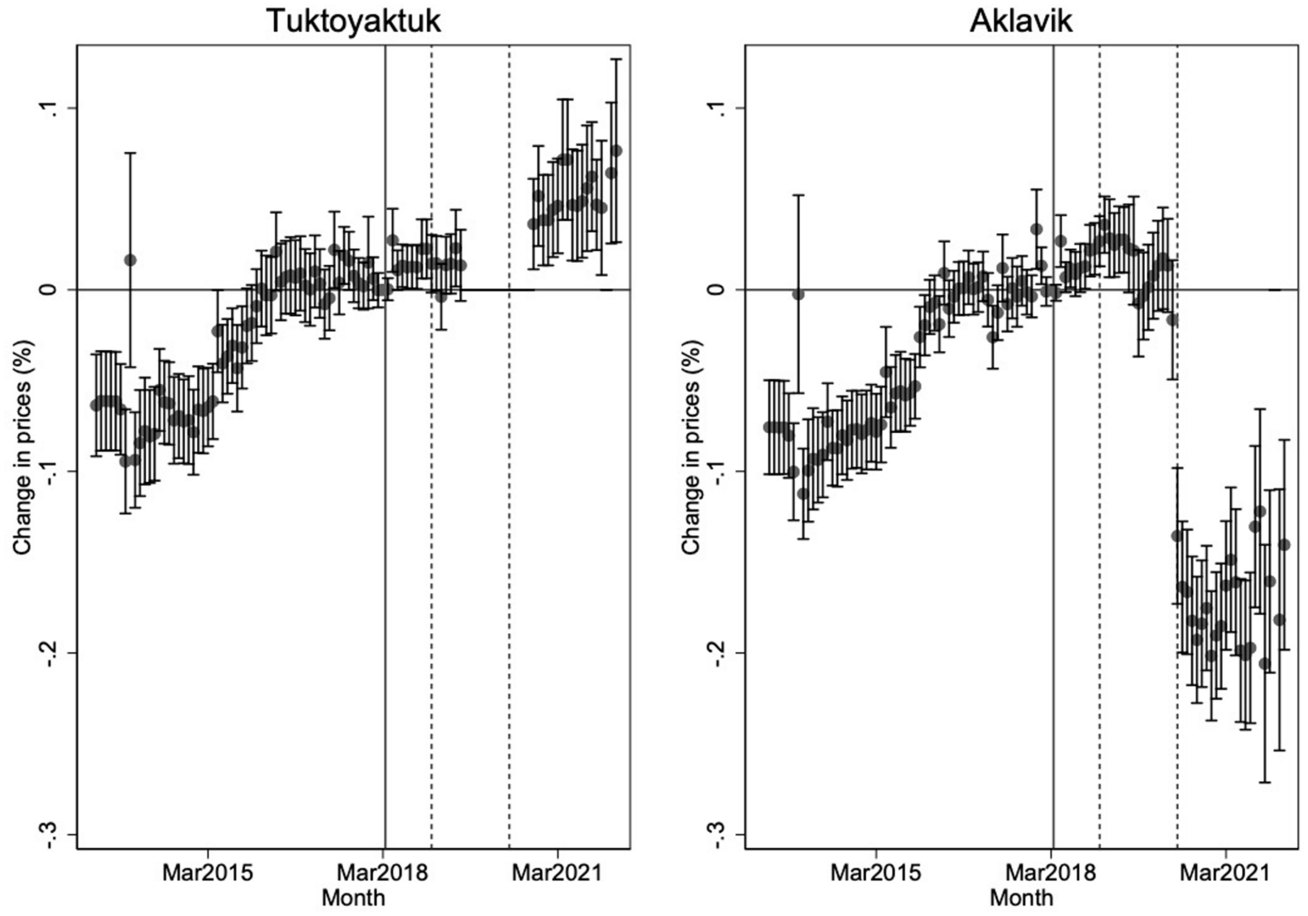
Change in own prices for each community in percent terms relative to March 2018. Vertical lines denote the end of subsidies in Tuktoyaktuk. The solid vertical line denotes the loss of NNC freight subsidy eligibility in Tuktoyaktuk and the two subsequent dashed vertical lines denote the timing of NNC subsidy increases for Aklavik in January 2019 and May 2020.

Finally, Figure 8 presents overall price trends by separately regressing log prices on time dummies for each community, using March 2018 as the baseline. The results show a significant long-term divergence in food prices, driven primarily by declining prices in Aklavik rather than rising prices in Tuktoyaktuk. By March 2022, prices in Tuktoyaktuk were approximately 7–8% higher than in March 2018, while prices in Aklavik were 12–15% lower. The largest price declines in Aklavik coincided with the May 2020 NNC subsidy increase, whereas the January 2019 subsidy increase had comparatively little immediate effect. Prices in Tuktoyaktuk showed modest upward drift in the first year after subsidy withdrawal, but major relative price increases became evident only after data reporting resumed in fall 2020.

## Discussion

This study assessed the economic impacts of the Inuvik-Tuktoyaktuk Highway and the concurrent withdrawal of NNC subsidies on food prices in Tuktoyaktuk. Informed by initial government projections and policy assumptions, three hypotheses were considered: (H1) that the combined effects of improved surface access and initial freight subsidy withdrawal would have a neutral or beneficial effect on food prices; (H2) that surface access would at least lower the cost of non-subsidized goods; and (H3) that the loss of access to future NNC subsidy enhancements would not significantly worsen food affordability.

Our findings allow us to critically assess the three hypotheses. For H1, we find that short-term projections of food price stability were roughly accurate, but longer-term data reveal that infrastructure improvements did not sufficiently offset the impacts of freight subsidy withdrawal. Retailer pricing behavior, including margin adjustments, may have contributed to gradual price increases, highlighting that changes in freight costs do not automatically translate into price changes for consumers. For H2, we find no evidence that surface access reduced the cost of non-subsidized goods, despite plausible expectations that improved trucking logistics and reduced emergency air freight needs would lower prices. This suggests that potential cost savings were either minimal or may not have been passed through, and casts doubt on the assumption that broader, unobserved product categories experienced substantial price improvements. Finally, relying on static cost assumptions failed to account for the community’s vulnerability to future freight subsidy changes and freight cost fluctuations, highlighting the need for adaptive policy frameworks that can respond to long-term uncertainties, rather than assuming infrastructure alone will ensure lasting affordability—thus rejecting our third hypothesis (H3).

### Net effects of highway access and freight subsidy loss (H1)

Proponents of the Inuvik-Tuktoyaktuk Highway argued that reduced freight costs from year-round road access would largely offset the effects of freight subsidy withdrawal, resulting in stable or even declining food prices. Our findings reject H1. Although the short-term effects (2018–2019) were negligible, suggesting that retailers initially absorbed some impacts, prices in Tuktoyaktuk rose sharply over the longer term. By March 2022, food prices had increased by nearly 20% relative to a counterfactual scenario in which Tuktoyaktuk had remained NNC-eligible. Thus, the highway’s anticipated cost-reduction benefits did not materialize to the extent needed to maintain food affordability.

### Impacts on non-subsidized goods (H2)

Even if overall prices were not lowered, it was reasonable to expect that improved surface access would reduce costs for non-subsidized goods, such as canned foods or dry goods previously reliant on seasonal ice roads or barge delivery, both through cheaper shipping and by minimizing reliance on costly emergency flights when inventories ran short. Our findings also reject H2. Analysis of individual food item prices showed no meaningful reductions in the price of non-subsidized goods, either in the short or long term. This suggests that freight cost savings, if they occurred, were not passed through to consumers. The highway failed to produce measurable benefits even for goods that should have been most affected by lower transport costs.

### Long-term effects of losing future subsidies (H3)

At the time of the highway’s construction, the risk of losing access to future enhancements to the NNC program was not clearly anticipated. Our findings demonstrate that this risk materialized and significantly amplified food cost disparities. After January 2019 and especially after May 2020, Aklavik received major freight subsidy increases for essential foods, while Tuktoyaktuk, no longer eligible, did not. As a result, relative food prices in Tuktoyaktuk increased sharply, with the largest disparities emerging for products that experienced the largest NNC subsidy boosts in Aklavik. This evidence rejects H3 and highlights the importance of accounting not only for future policy changes but also for uncertainty in future freight costs across different modes in prospective infrastructure planning. As with air freight costs, surface freight costs are vulnerable to fluctuations driven by fuel prices, climate-related disruptions, and evolving supply chain dynamics, all of which can reshape the economics of northern food systems over time. Infrastructure planning and food security strategies must therefore adopt a more dynamic, risk-aware approach that recognizes the complex and shifting factors influencing food access in remote Indigenous communities. Our results reinforce the need for adaptive, shock-responsive policy design. Infrastructure investments must be accompanied by mechanisms that monitor ongoing cost dynamics and provide responsive supports when needed, particularly to safeguard food security in an increasingly volatile northern environment.

### Broader interpretation

Our findings point to a critical divergence between infrastructure promises and real-world outcomes. Although the Inuvik–Tuktoyaktuk Highway was expected to improve food affordability by reducing freight costs, transportation improvements alone were insufficient to counteract the withdrawal of NNC subsidies. In fact, food prices in Tuktoyaktuk rose by nearly 20% relative to a counterfactual scenario in which the community had remained eligible for NNC subsidies, like Aklavik.

Household production theory (17) suggests that improved infrastructure may reduce the “full price” of food by lowering the time and effort required for consumer travel, thereby increasing demand in destination markets such as Inuvik. However, this mechanism appears unlikely to explain the price increases observed in Tuktoyaktuk. The community, home to just over 1,000 residents, is connected to Inuvik by a 138-kilometre remote highway with no services along the route. Travel requires winterized vehicles, significant fuel expenditures, and several hours of round-trip driving—barriers further compounded by caregiving responsibilities, employment obligations, and seasonal conditions. These structural constraints likely limit the feasibility of regular food shopping outside the community. Preliminary insights from ongoing community-based research, including qualitative interviews and a household cost-of-living survey (Slack et al., in preparation), suggest that while some discretionary travel occurred shortly after the highway opened—a “novelty effect”—this behavior did not persist. Food purchasing appears to remain largely localized, and the expansion of consumer choice may be occurring through online retailers rather than through routine physical travel. Although results are forthcoming, these early indications suggest that changes in consumer mobility have been limited during the study period.

These findings support the interpretation that the observed food price increases in Tuktoyaktuk were not driven by increased consumer access or demand displacement, but rather by structural shifts in freight subsidy policy and retail pricing behavior. In the short term, stores in Tuktoyaktuk appear to have mitigated the impact of freight subsidy reductions by selectively lowering prices for subsidized goods, while maintaining higher prices on unsubsidized items. This strategy resulted in minimal initial price increases and reflects a very low pass-through of freight subsidy reductions (27). However, over time, products that experienced the greatest loss of subsidies compared to Aklavik saw the largest relative price increases, revealing deeper structural challenges in maintaining food affordability without freight subsidy support. These pricing dynamics were further compounded by long-term shifts in subsidy policy that disproportionately affected Tuktoyaktuk. Significant changes to the NNC program, including targeted subsidy increases for essential foods in January 2019 and broader enhancements in May 2020 (due to COVID-19), were implemented for communities like Aklavik but not for Tuktoyaktuk, which had been phased out of eligibility. These adjustments helped moderate or even decrease food prices in Aklavik, while Tuktoyaktuk faced escalating costs without comparable supports, amplifying relative disparities over time.

The cumulative financial impacts of these changes were substantial, underscoring the heavy burden placed on households following the withdrawal of subsidies. Although the Inuvik–Tuktoyaktuk Highway was expected to reduce food costs by improving transportation access, it did not prevent a sharp rise in food expenses. By 2019—the last year for which we have participatory food costing data prior to the COVID-19 pandemic—the Revised Northern Food Basket (RNFB) cost for a family of four in Tuktoyaktuk reached CAD$23,253 annually, representing approximately 30% of household income, compared to just 10% in Ottawa (28).

Prior to freight subsidy withdrawal, the NNC program had provided an average of approximately CAD$327,000 annually to food retailers in Tuktoyaktuk, equivalent to over CAD$300 per capita—or roughly CAD$1,200 per family of four residents (29, 30). However, following the removal of these subsidies, food costs rose sharply. Participatory food costing suggests an 8.68% increase in the RNFB between spring 2018 and winter 2019, translating into an additional annual burden of approximately CAD$2,013 per family of four—a financial burden 1.68 times greater than the value of the former freight subsidy. This shift effectively transferred the cost burden from the public system onto individual households, who are now paying directly for food costs that were previously subsidized. Not only did families lose vital financial support, but they also faced even higher costs than before, deepening the financial strain on an already food-insecure community.

These findings highlight the risks of assuming that infrastructure improvements alone can address the complex drivers of food insecurity in northern communities. Without sustained investment across multiple systems—including subsidies, food governance, transportation supports, and community-led food security initiatives—the economic challenges faced by northern Indigenous communities may worsen rather than improve.

Previous literature has consistently highlighted positive economic outcomes associated with highway development, with studies showing enhanced economic prospects following road construction (31–33). Impact assessments in the Canadian North similarly anticipated economic benefits from projects like the Inuvik–Tuktoyaktuk Highway (18, 34–36). These studies presented a hopeful outlook that improved transportation access would reduce the cost of living, including food costs, for remote communities (8).

However, our findings complicate this narrative. Despite anticipated transportation cost savings, the withdrawal of NNC subsidies ultimately outweighed any benefits from improved road access. As a result, food prices in Tuktoyaktuk rose sharply, highlighting a significant gap between governmental promises to enhance food security for Indigenous communities (37) and the lived experience of households. The removal of freight subsidy supports occurred amidst persistent food insecurity, without clear evidence that infrastructure alone could close the affordability gap. This underscores a broader disconnect between policy intentions and on-the-ground outcomes.

Critiques of the NNC program have long emphasized concerns regarding eligibility criteria (38). Although the program’s budget was expanded in 2016 to include more communities and raise freight subsidy levels, its persistent exclusion of communities with road access—but located far from distribution centers—reflects a major oversight. Road connectivity alone does not eliminate the logistical and cost challenges associated with supplying northern food systems. As our findings show, infrastructure metrics are an insufficient basis for food policy decisions. To meaningfully address food insecurity, eligibility criteria and program designs must be grounded in robust evidence, community realities, and Indigenous knowledge systems (39).

Furthermore, our study underscores the need for ongoing monitoring and evaluation of living conditions in the North, particularly following major infrastructure or policy shifts. Reliance on short-term data can obscure long-term impacts, as shown by the evolving food price disparities between Tuktoyaktuk and Aklavik. Establishing a framework for continuous assessment of food prices, living costs, and food security indicators is critical to ensure that the benefits of infrastructure investments are sustained—and that emerging challenges are identified early.

Finally, our findings point to the urgent need to improve transparency and access to food pricing data in remote northern communities, where information is often proprietary and difficult to obtain (11, 40, 41). Reliable, accessible data is fundamental for supporting effective policy analysis, community planning, and advocacy. Addressing these structural barriers is key to building equitable, community-driven food systems that are resilient to future disruptions.

### Limitations

Our analysis is subject to a few important limitations. First, we lack access to retailer transport and inventory cost data, which limits our ability to precisely attribute observed price dynamics to specific operational changes within Tuktoyaktuk’s retail sector. It remains unclear whether transportation costs decreased following the highway’s construction, and if so, to what extent any savings were passed on to consumers. This is particularly relevant given previous findings on NNC subsidy pass-through (27), which suggest that while subsidy pass-through is mandated under program rules, transport cost pass-through is voluntary and may not be systematically reflected in consumer prices. Second, our dataset covers only a subset of food products, primarily focusing on items included in the RNFB. Broader shifts in retail pricing strategies following the end of NNC eligibility—potentially affecting both subsidized and unsubsidized goods—may not be fully captured. Thus, while our findings reveal significant trends, they do not represent the entire retail food environment. In addition, while this study documents important direct changes in food costs, it does not fully account for broader economic dynamics that may influence food affordability. These include potential changes in purchasing power, household income, employment opportunities, and local retail competition—all factors that could have been impacted by the introduction of year-round surface access.

Finally, the critical role of country foods in Inuit food systems—central to nutrition, culture, and community well-being—was beyond the scope of this study’s economic assessments. However, the relationship between infrastructure development, harvesting practices, and access to country foods warrants greater attention. Improved transportation access may influence travel patterns, food purchasing behaviors, and harvesting activities, with complex effects on food security and dietary practices. Future research should examine how infrastructure and policy changes impact both the market food economy and the traditional food economy, to better capture the full spectrum of consequences for Indigenous food security, sovereignty, and resilience (27).

### Implications and significance of the study

Despite certain limitations, this study provides critical insights for infrastructure planning, food policy development, and broader efforts to strengthen food security in northern and Indigenous communities. This research offers several methodological strengths, notably its community-determined and co-produced approach, which was instrumental in defining the research questions and accurately documenting food costs. The participatory food costing methodology, rooted in local engagement, provides robust and contextually relevant insights into economic conditions before and after major infrastructural changes.

Our findings underscore the necessity of ongoing monitoring and the development of dynamic, responsive policymaking—particularly regarding food security and economic stability in remote areas. The unanticipated rise in food prices, despite substantial transportation investments, highlights the need for policies that are informed by ground realities and that are adaptable to actual outcomes rather than projected benefits. Infrastructure projects must be coupled with coordinated, sustained supports to avoid inadvertently exacerbating existing vulnerabilities. More specifically, this research has important implications for future infrastructure projects across the Canadian North and similar contexts globally. While the Government of Canada has committed over CAD$400 million to new northern transport infrastructure initiatives, our findings suggest that transportation improvements alone are insufficient to address complex issues such as food affordability (42). The case of Tuktoyaktuk illustrates that infrastructure without concurrent supports—such as subsidies, food governance initiatives, and culturally appropriate food security programs—risks transferring financial burdens from the public sector onto individual households. This reality must be integrated into future policy planning.

The Government of Canada’s investments, including projects like the Tłı̨chǫ All-Season Road (opened in 2021) and the proposed NWT Mackenzie Valley Highway and Canadian Northern Corridor, aim to enhance connectivity and reduce costs for northern communities (35, 43). However, the actual impacts of these projects on local food prices, household economies, and food security conditions require ongoing, careful monitoring. Our study highlights that an Indigenous-determined, co-conducted, evidence-based approach is essential to ensure that infrastructure investments not only promote economic growth but also tangibly improve food security and well-being in northern and Indigenous communities.

More broadly, these findings emphasize the need for systemic, multi-sectoral approaches to food security in northern regions. As emphasized in the Inuit Nunangat Food Security Strategy (13), food security is shaped by the interplay of governance, logistics, operations, management, and policy. Infrastructure investments must therefore be integrated with actions across sectors—including supports for harvesting, retail governance reforms, improved transportation logistics, and initiatives to strengthen Inuit food sovereignty—to meaningfully address persistent food insecurity. The experience of Tuktoyaktuk serves as a cautionary example of the risks associated with focusing narrowly on infrastructure solutions without simultaneously reinforcing the broader systems that underpin food access, affordability, and community resilience.

## Conclusion

The Inuvik–Tuktoyaktuk Highway, despite its anticipated benefits, has not sufficiently alleviated high food costs in Tuktoyaktuk. Our analyses demonstrate that, following the withdrawal of NNC subsidies, food prices in Tuktoyaktuk rose by nearly 20% by March 2022 relative to a counterfactual scenario in which freight subsidy eligibility had been maintained. These findings underscore the complexity of infrastructure impacts on local economies, particularly in remote Indigenous communities, where transportation improvements alone are insufficient to secure food affordability. The significant economic repercussions observed post-highway completion highlight the critical need for integrated policy approaches that align infrastructure investments with sustained support measures, such as food subsidies, food governance reforms, and community-led food security initiatives. Without such coordinated action, well-intentioned infrastructure projects risk exacerbating economic burdens rather than alleviating them. Moving forward, it is imperative that policymaking be grounded in robust, community-informed research that captures the interconnected dimensions of food security, transportation access, and economic resilience. Our findings emphasize the importance of ongoing monitoring, continuous evaluation, and adaptive policy frameworks that are responsive to the lived realities of northern and Indigenous communities—ensuring that future infrastructure investments genuinely contribute to well-being, equity, and food security.

## Data Availability

The original contributions presented in the study are included in the article/supplementary material, further inquiries can be directed to the corresponding author.
